# Assessing Emotions of Teaching Assistants in Inclusive Education

**DOI:** 10.3389/fpsyg.2022.813726

**Published:** 2022-07-14

**Authors:** Lan Yang, Chia-Ling Hsu, Tianfang Ye, Kuen Fung Sin

**Affiliations:** ^1^Department of Curriculum and Instruction, The Education University of Hong Kong, Tai Po, Hong Kong SAR, China; ^2^Hong Kong Examinations and Assessment Authority, Wan Chai, Hong Kong SAR, China; ^3^Department of Applied Social Sciences, The Hong Kong Polytechnic University, Kowloon, Hong Kong SAR, China; ^4^Department of Special Education and Counselling, The Education University of Hong Kong, Tai Po, Hong Kong SAR, China

**Keywords:** teaching assistants (TAs), inclusive education, teacher emotion inventory (TEI), teacher emotions, validation, the TAs’ emotion model, Hong Kong

## Abstract

Although there are an increasing number of studies on assessing teacher emotions in mainstream education, there is a lack of appropriate measurement tools to evaluate the emotions of teaching assistants (TAs) who need to take care of students with a range of special educational needs (SEN). This study tested the generalizability of the 24-item teacher emotion inventory (TEI), among 204 TAs from 122 secondary schools with inclusive education in Hong Kong. We conducted both confirmatory factor analysis (CFA) and Rasch analysis to test the within-network validity of the TEI. For the between-network validity, we examined the relationships between TAs’ emotions and their attitude toward inclusive education. The Rasch analysis supported the scale’s dimensionality and item fit statistics. The CFA supported the five-factor solution of the TEI. The results also showed statistically significant correlations between positive emotions (joy and love) and TAs’ attitudes toward inclusive education. TAs’ negative emotions (anxiety, anger, and stress) appeared to be negatively correlated with their attitude toward inclusive education. The results supported that TEI is a useful tool to assess the emotions of TAs that play a pivotal role in assisting both school teachers and SEN students, who are more likely to face increased emotional challenges than those not needing to educate SEN students. Implications of this study to enrich the current scope of research on understanding teacher emotions across educational levels and settings are discussed.

## Introduction

In the past two decades, research on teachers’ emotions has continuously called for increased attention, emphasizing its importance and regarding teachers’ emotions as “the heart” of teaching and teachers’ lives (e.g., Hargreaves, 2001; Frenzel, 2014). Teacher emotions, as updated by Frenzel et al.’s (2019) definition, are the “emotions experienced in the context of their professional engagement as teachers” (p. 2). Moving to define emotions specifically, Frenzel et al. (2019) conceptualized emotions as “multicomponential constructs” that are based on the theoretical conceptualization of emotions by Fontaine et al. (2007, p. 1050) “as consisting of variably interrelated changes in activity across a set of six components [e.g., subjective experiences (feelings), appraisals of events, action tendencies, etc.]” [refer also to Scherer (2005)]. The multicomponential features of emotions, namely, discrete positive emotions (e.g., enjoyment and pride) and negative emotions (e.g., anger, anxiety, shame, and boredom), have been widely explored for around a decade. Consistently, researchers discovered that teachers’ emotions play a remarkable role in students’ learning and academic success (Pekrun and Linnenbrink-Garcia, 2014; Frenzel et al., 2016). Frenzel et al. (2019) found that teachers’ enjoyment enhances students’ enjoyment, being that this relationship is partially mediated by teachers’ enthusiasm. In a recent review by Chen (2021), teachers’ emotions also affect their self-efficacy, engagement, wellbeing in teaching, and job satisfaction. Taxer and Frenzel (2015) surveyed 266 teachers and found that their expressive, positive emotions (e.g., happiness, pride, and enthusiasm) were significantly correlated with their wellbeing, relationship with students, and teaching efficacy.

While studies on teacher emotions in Western countries are growing, Lee and Yin (2011) argued that “there is a remarkable dearth of such studies in China” (p. 25). Based on in-depth qualitative inquiries among 25 Chinese teachers, Yin and Lee (2012) summarized four emotional rules of Chinese teachers, namely, maintain positive emotions for the purpose of making students happy in their learning; instrumentalize emotions for motivating students to learn and achieve teaching goals; control negative emotions in teaching, trying to avoid possible disturbing effects on teaching and learning; and stay passionate for teaching commitments (pp. 60–61). Chen (2019a) extended the work by Yin and Lee to pre-service teachers and in-service primary and secondary schools teachers (Chen, 2016, 2019b,c) by using quantitative approaches to assess both positive emotions (e.g., love and joy) that are consistent with the emotional roles identified by Yin and Lee (2012), and negative emotions (e.g., anger and sadness) that teachers experience in their work (despite the fact that teachers may control their negative emotions and do not express them to their students). Although emotion research on mainstream Chinese teachers has drawn increasing attention and efforts, research on teachers who teach students with special educational needs (SEN) or teaching assistants (TAs) who need to support school teachers to teach SEN students in inclusive education remain under-researched. Inclusive education in this study refers to “a process of strengthening the capacity of the education system to reach out to all learners and can thus be understood as a key strategy to achieve Education for All” (UNESCO, 2009, p. 8). Since 1997, the Hong Kong government has also launched a series of supporting policies to support the development of inclusive education (Education Department, 2002; Education Bureau, 2008), including financial support to schools to recruit TAs (Education Bureau, 2012). Given their specific and highly demanding duties to teaching and caring for SEN students, it is of great importance to study their emotional experiences in inclusive education for developing tailor-made training and support. The following sections review the existing literature on emotion research.

## Literature Review

### Emotions of Teachers Who Teach Special Educational Needs Students

In a systematic review by Yang et al. (2021) based on 103 studies, only 4 (out of the final 38 screened out studies) were conducted among teachers who teach students with SEN (Mak et al., 2004; Kaufhold et al., 2006; Ruble et al., 2011; Chong and Leung, 2012). Chong and Leung (2012) found that teachers who teach SEN students are more likely to have negative thoughts and a stronger sense of burnout, leading to a higher turnover rate. Consistently, among over 228 special educators in South Texas schools, Kaufhold et al. (2006) found that teachers’ sense of burnout is caused by a lack of supplies or materials supporting SEN students. In the setting of Hong Kong, Mak et al.’s (2004) study found insufficient support and training to these supporting staff who take care of children with developmental disabilities, further leading to job dissatisfaction. Ruble et al. (2011) found special education teachers experienced emotional exhaustion in teaching children with autism spectrum disorder. While the existing literature shows these positive functions in teaching (e.g., quality of teaching, teacher identity development, job satisfaction, and wellbeing) are associated with teachers’ positive emotions (e.g., Zembylas, 2003; Chen, 2016; Wang et al., 2019), research conducted on teachers who need to teach and support SEN students is sparse, let alone research conducted on TAs’ emotions.

In search of validated instruments to assess the emotions of special school teachers in Hong Kong, Yang et al. (2021) conducted a systematic review and found six validation studies (Chen, 2016; Frenzel et al., 2016; Hong et al., 2016; Burić et al., 2019; Gramipour et al., 2019; Atmaca et al., 2020). Emotions assessed in these validated instruments are similar (refer to Supplementary Appendix Table 1). Considering both the length and previously accumulated validation evidence on Chinese teachers (Chen, 2019a,c), Yang et al. (2021) extended Chen’s (2016) teacher emotion inventory (TEI) to Hong Kong teachers and provided initial validation evidence on special education teachers whose main duties are to cater to the needs of students who have moderate-to-severe SEN. In this study, we consistently used the TEI developed by Chen (2016) and examined by Yang et al. (2021) in Hong Kong special education among another sample of TAs.

### Emotions of Teaching Assistants in Inclusive Education

There has been an increase in TA positions in schools in the United Kingdom and the United States (Webster and Blatchford, 2012, 2013; Giangreco, 2013). As highlighted by Webster and Blatchford (2013), the increased numbers of TAs in the United Kingdom are due to the popular trend of inclusive schools. Students with SEN and physical/mental disabilities can study with typically developed students in inclusive classrooms. The second reason is that TAs can reduce teachers’ workload in inclusive education. There has also been an evitable trend to hire TAs in Hong Kong (Rose and Forlin, 2010), given the increasing number of SEN students who are integrated into mainstream schools. The Education Bureau (EDB) in Hong Kong has subsidized schools to recruit more TAs to support SEN students (Education Bureau, 2012). It is surprising to find “the number of teaching assistants (TAs) working in mainstream schools has soared in recent years as students with special educational needs (SEN) are integrated into regular classrooms. However, research on TAs is rare” (Chan et al., 2020, p. 1).

In our systematic review (Yang et al., 2021) on the measurement of teacher emotions and extended research on special education teachers, we found three research articles (Rose and Forlin, 2010; Giangreco, 2013; Graves, 2014) and three doctoral/master’s theses/dissertations (Chilton, 2012; Heslop, 2012; Kipfer, 2015) that reported secondary observations of TAs’ emotions while supporting inclusive or SEN classes in mainstream schools. The study by Giangreco (2013) brought attention to the current shortcomings of inclusive schools catering to SEN students in the United States context. It criticized the overuse and misuse of teacher assistants as a symptom in the United States context: “Too often teacher assistants are not used wisely in inclusive classrooms, but rather metaphorically as a band-aid for an injury that at the least requires stiches and possibly major surgery; no band-aid, regardless of size or type, will meet the need” (Giangreco, 2013, p. 94).

The research conducted by Rose and Forlin (2010) did not explicitly study TAs’ emotions as a focus. Still, TAs reflected that professional training helped their growth, confidence levels, competence, feeling of empowerment, and feeling more prepared to fulfill their job responsibilities. The doctoral thesis written by Heslop (2012) indicated some secondary reflections by TAs who felt supported when they were “being valued, included, and involved” in schools. They also reported that their “relationships with other support staff and school staff” (p. iii), and guiding beliefs of the school contributed to their positive feelings. Kipfer (2015) highlighted the following three themes about the needs of education assistants (EAs) in Canada: (1) a need to collaborate with class teachers to be able to effectively support the transition; (2) a need to get trained to update their programming skills to teach SEN students; and (3) having a good relationship with the class teachers to feel valued. Chilton (2012) highlighted that learning support assistants (LSAs) felt they were appreciated by the class teachers who they worked with. LSAs also felt valued due to the contribution that they made to students’ learning. Chilton (2012) also highlighted that LSAs’ expressed their feelings of happiness and their experience of being rewarded for their role in school when they see smiling students.

#### Teaching Assistants’ Negative Emotions

Rose and Forlin’s (2010) study mentioned TAs’ feelings of frustration due to unclear job specifications assigned to them. Graves’s (2014) study reported that TAs felt they were being exploited and mistreated in terms of earning less pay and required to work on their day-off compared to the teachers. They also expressed that their role was “poorly understood and their contribution was not recognized” (p. 261) by the senior officials. However, despite such negative feelings expressed by some participants in Graves (2014), this study did not explore specific negative emotions as documented in previous research (e.g., stress, anger, anxiety, and hopelessness). Kipfer (2015) reported that TAs felt valued by their students; however, “TAs did feel appreciated by the student, but it was clear that many did not feel appreciated by the classroom teacher” (p. 27).

#### Consequences of Teaching Assistants’ Emotions

Rose and Forlin (2010) reported positive feelings of TAs due to the training provided, such as growth in confidence levels, feeling of empowerment, and sense of more preparedness in performing their job duties, which seems to positively help TAs practices in supporting inclusive and SEN classrooms. Heslop’s (2012) study found that TAs’ feelings of “being valued, included and involved” positively contribute to their practices (p. iii). Similarly, Chilton’s (2012) study suggested that TAs’ feeling of being appreciated by the class teacher may promote better outcomes. Consistently, Kipfer (2015) found that TAs’ good relations with other staff members also positively helped the practice of TAs. As for TAs’ negative feelings in inclusive education, Rose and Forlin (2010) in the Hong Kong context and Graves (2014) in the context of the United Kingdom (England) shared that some of the negative feelings experienced by TAs (e.g., disrespected, low morale, and frustrated) influenced their performance negatively. These studies suggested that the scarcity of research studies predominantly aimed at exploring the impacts of TAs positive or negative emotions on their practices in inclusive or SEN settings.

### Emotions of Teaching Assistants: What’s Next?

Although the above-reviewed studies mentioned TAs’ emotions slightly or moderately, none of them were designed to study TAs’ emotions *per se*. No such study has explicitly considered using a quantitative research design with validated instruments to assess TAs’ emotions while teaching/supporting SEN students and the associations of these emotions. These associations may cover essential variables in inclusive education [e.g., TAs’ attitudes toward inclusive education (AIE) or TAs’ self-efficacy in supporting SEN students]. For example, Graves’s (2014) study in the United Kingdom used “fifteen face-to-face and individualized semi-structured interviews through email,” and “three asynchronous online focus groups” (p. 259). Rose and Forlin’s (2010) study focused on delivering an intervention of professional development training for TAs in Hong Kong using mixed methods through pre- and post-surveys with MCQs and open-ended questions, reflective texts, and focus groups. Heslop (2012) used mainly qualitative methods (e.g., focus groups and semi-structured face-to-face interviews) for data collection from TAs, senior managers, and educational psychologists in United Kingdom schools. Kipfer (2015) used a qualitative method of conducting semi-structured interviews in Canadian schools. Chilton’s (2012) study involved a semi-structured individual interview and group interview of LSAs and observations of four classes in a United Kingdom school. Taken together, none of them used any multidimensional instrument to assess TAs’ discrete emotions. On these methodological limitations, Frenzel et al. (2016) commented: “It is striking that much of the current scientific knowledge on discrete teacher emotions rests on narrative, sometimes single case data, whereas quantitative data are scarce” (p. 148). To address the research gap, this study used the TEI that has been extended from previous research (Chen, 2016) to special education teachers (Yang et al., 2021) in order to measure the discrete emotions of TAs.

### The Conceptual Framework of This Study

This framework has its theoretical origin in the control-value theory of achievement emotions (Pekrun, 2006). It was recently extended to the population of teachers to form the teacher emotion model (TEM; Chen, 2021). Based on the studies by Pekrun (2006) and Chen (2021), Yang et al. (under review)^1^ developed a modified version in relation to the inclusive education context in order to examine the relationship between TAs’ self-efficacy and their emotional wellbeing. Based on this conceptual framework, we also included a subscale to assess TAs’ AIE.

The two key research purposes were to test (1) the within-network validity of the TEI (Yang et al., 2021) with a sample of TAs who needed to support SEN students and (2) the between-network validity by examining the relationship between TAs’ emotions and their AIE.

The following two fundamental hypotheses (Hs) were examined in this study:

*Within-network validity: item- and factor-level analyses of TAs’ emotions*

H1: The five-factor solution of TAs’ emotions (i.e., love, joy, anger, stress, and sadness) will be the best compared to the one general factor solution and the two-factor solution.

*Between-network validity: relating TAs’ emotions to AIE*

H2: Correlations between the two positive emotions (love and joy) and TAs’ AIE will be significantly positive. Comparatively, correlations between the three negative emotions (anger, sadness, and stress) and TAs’ AIE will be significantly negative. A further path analysis will also provide Supplementary Information to the relations between TAs’ emotions and AIE.

## Materials and Methods

### Participants and Procedure

The participants were 204 TAs (29% male) from 122 secondary schools of Hong Kong. The data collection was conducted in November 2020. Given that there were 392 public sector secondary schools documented in October 2020 (GovHK, 2021)^2^, at least one TA represented one of the 122 secondary schools to attend a professional training workshop commissioned by Education Bureau (EDB) to the Centre for Special Educational Needs and Inclusive Education (CSENIE). This sample of TAs represented 31% of local secondary schools. The majority of them (87%) were from 20 to 29 years old. All of them responded to the 24-item TEI scale (Yang et al., 2021) and the 4-item scale of AIE (CSENIE, 2010). The research team achieved ethical approval from Human Research Ethics Committee (HREC) at The Education University of Hong Kong (EdUHK) before the data collection commenced.

### Measures

#### Teacher Emotion Inventory

The TEI contains five subscales that were adopted to assess TA emotion in inclusive schools: love (4 items), joy (6 items), anger (4 items), sadness (4 items), and stress (6 items). The psychometric properties of the 24-item TEI have been investigated in a sample of SEN teachers of Hong Kong *via* Rasch analysis, and all five subscales had satisfactory reliabilities (Yang et al., 2021). Especially, the Cronbach’s α values were greater than 0.7 (i.e., love = 0.82, joy = 0.77, anger = 0.87, sadness = 0.82, and stress = 0.83). All items in the five subscales were rated on a four-point rating scale, with response categories ranging from strongly disagree (1) to strongly agree (4). A higher score for each subscale reflected stronger emotion for TAs working in inclusive schools.

#### Attitude Toward Inclusive Education

We used the four-item scale for measuring TAs’ AIE. This scale was developed and validated by the Center CSENIE. The Cronbach’s α values for primary (*n* = 640) and secondary school teachers (*n* = 425) were 0.83 and 0.85, respectively (Sin et al., 2012). The Cronbach’s α value of this scale used with a sample of 586 parents of SEN students in inclusive education was 0.80 (Lui et al., 2015). The TAs were rated on a four-point rating scale ranging from 1 = strongly disagree to 4 = strongly agree. A sample item was “I think inclusive education can provide equal learning opportunities for SEN students.” A higher AIE score indicated TAs’ more positive AIE for students with SEN. The Cronbach’s α value was 0.83 in this study.

### Data Analysis

First, we utilized two analytic approaches, namely, confirmatory factor analysis (CFA) and Rasch analysis, to assess the two scales’ qualities with a sample of TAs working in special schools. Such a mixed analysis method has been widely used in many empirical studies (e.g., Deneen et al., 2013; Hart et al., 2013; Yan, 2016), which can provide ample pieces of evidence of the psychometric properties of an instrument. CFA can primarily provide an overall test-level evaluation (at the macro level) from a global model-data fit. Rasch analysis can mainly supply each item-level examination (at the micro level) *via* item fit statistics. The one general factor, two-factor, and five-factor structures of the TEI were examined in detail. Then, bivariate correlations between the TEI and the four-item AIE scale were calculated in order to present their relations.

#### Confirmatory Factor Analysis

Confirmatory factor analysis was used to examine the global model-data fit for the one-, two-, and five-factor structures of the TEI at the test level. CFA was conducted *via* the R package lavaan (Rosseel, 2012). No cross-loading or correlated residual was specified, the variance of each latent factor was fixed to 1, and covariances between latent factors were free to be estimated for the measurement model. Fit indices used to check the model-data fit were the comparative fit index (CFI), standardized root mean square residual (SRMR), root mean square error of approximation (RMSEA), and model χ^2^ test statistics (Steiger, 1990; Hu and Bentler, 1999). As a rule of thumb, values of CFI greater than 0.90, and values of SRMR and RMSEA smaller than 0.08 are considered as having acceptable model-data fit (Kline, 2016).

#### Rasch Analysis

Rasch analysis was employed to evaluate the psychometric properties of the three models at the item level. Rasch analysis’s rationale was used to check the degree to which an empirical data fit (or satisfied) a prespecified model. Rasch analysis uses a mathematical equation for the relation of the probability of an item’s correct response to the underlying latent construct(s). This study adopted the multidimensional random-coefficients multinomial logit model (MRCMLM; Adams et al., 1997) using the R package TAM (Robitzsch et al., 2020) to assess the one-, two-, and five-dimensional models, respectively.

In the MRCMLM, the probability of a response in category *k* of item *j* for person *i* with latent traits (constructs) θ is defined as follows:


(1) $$p_{\text{ijk}}=\frac{\exp\left(b_{\text{jk}}^{\text{T}}\,\theta_{\text{i}}+a_{\text{jk}}^{\text{T}}\,\xi \right)}{\sum_{k=1}^{K_{j}}\exp\left(b_{\text{jk}}^{\text{T}}\,\theta_{\text{i}}+a_{\text{jk}}^{\text{T}}\,\xi \right)}$$

Where *K*_j_ is the number of categories in item *j* (i.e., *K*_j_ = 4 for every item in this study), ξ is a vector of difficulty parameters that describes the items, *b*_jk_ is a score vector given to category *k* of item *j* across the latent traits, and *a*_jk_ is a design vector given to category *k* of item *j* that describes the linear relationship among the elements of ξ. Using *a*_jk_ and *b*_jk_ to define the relationship between items and persons enables the MRCMLM to include the most unidimensional and multidimensional Rasch-based models, such as the Rasch model (Rasch, 1960), the rating scale model (RSM; Andrich, 1978), and the partial credit model (Masters, 1982). As the items in the TEI are assumed on the same kind of rating scale, the unidimensional and multidimensional forms of the RSM in the MRCMLM were selected to fit the data.

For the TEI scale, to select the most fit model from the three competing models (with one-, two-, and five-factor solutions), we employed model-fit statistics such as Akaike’s information criterion (AIC; Akaike, 1987), Bayesian information criterion (BIC; Schwarz, 1978), and consistent AIC (CAIC; Bozdogan, 1987). For two comparative models, the model with lower values of AIC, BIC, and CAIC indicated a better fit between the model and data. Then, item fit indicators including response category functioning and item fit statistics [i.e., infit and outfit mean square error (MNSQ) statistics] were used to check the two scales’ qualities. The MNSQ values in the range of 0.6–1.4 represent a good fit between data and model (e.g., Wright et al., 1994; Linacre, 2006).

## Results

### Confirmatory Factor Analysis

Table 1 shows the CFA results for the TEI. The one-factor model for all 24 items was not a good fit according to the values of the CFI, RMSEA, SRMR, and robust χ^2^. Standardized factor loadings ranged from 0.08 to 0.90. Around half of the items had non-significant factor loadings (*p*-values > 0.05). The two-factor model categorizing the five emotions into positive and negative dimensions had better model-fit indices (CFI = 0.73) compared to the one-factor model (CFI = 0.33). In line with previous research (e.g., Chen, 2016; Yang et al., 2021), the five-factor model for classifying the 24 items into five factors had adequate model fit [i.e., CFI = 0.86, RMSEA = 0.09, SRMR = 0.08, and robust χ^2^ (242) = 540.05]. All standardized factor loadings were significant (*p*-values < 0.001) and ranged from 0.54 to 0.92. Yet, the modification indices suggested several items had correlated residuals. We read that the content of these pairs of items suggested correlations in the first CFA and found two justifiable reasons in light of previous research (e.g., Cole et al., 2007) to correlate three pairs of items: these items were designed to assess the same emotion and these items shared similar meanings (e.g., both items 1 and 2 of joy described positive feelings generated from working with school colleagues). As such, we performed the second CFA for the five-factor model, wherein three pairs of correlated residuals in joy, sadness, and anger were added. The modified five-factor model demonstrated an improved model fit [CFI = 0.90, RMSEA = 0.07, SRMR = 0.08, and robust χ^2^ (239) = 444.84]. Consequently, the modified five-factor model was selected as the preferred model. The standardized factor loadings are presented in Table 2. The latent factor correlation between love and joy was 0.57, *p* < 0.001, between sadness and anger was 0.86, *p* < 0.001, between stress and sadness was 0.71, *p* < 0.001, and between anger and stress was 0.57, *p* < 0.001. Correlations between other variables were non-significant.

**TABLE 1:** Confirmatory factor analysis and model fit indices of the TEI.

Models	CFI	RMSEA	SRMR	Robust χ^2^
One-factor solution	0.33	0.16	0.22	1638.16
Two-factor solution	0.73	0.10	0.09	806.80
Five-factor solution (a)	0.86	0.08	0.08	540.050
Five-factor solution (b)	0.90	0.06	0.07	444.840

*Five-factor solution (b) = Minor correlated residuals in joy, sadness, and anger were added based on their conceptual content; See also Figure 3.*

**TABLE 2:** Summary of Rasch analysis for the TEI under the five-dimensional model.

Subscale/Item	Standardized Factor Loadings	Item Difficulty*	SE	Infit MNSQ	Outfit MNSQ
**Love**					
(1) I love the profession of teaching assistant because unlike other professions, I can witness the growth and development of students with special educational needs.	0.54	1.590	0.143	0.88	1.04
(2) I love the profession of teaching assistant, because this profession can be respected and recognised by the society.	0.85	−1.637	0.159	1.04	1.00
(3) I love the profession of teaching assistant, because it makes me feel stable.	0.76	−1.687	0.159	0.99	0.93
(4) I love the profession of teaching assistant because the salary is reasonable.	0.61	0.090	0.145	1.49	1.57
**Joy**					
(5) I am motivated by support from my colleagues and leaders.	0.46	−1.953	0.158	0.94	0.95
(6) I enjoy sharing with my colleagues.	0.63	−2.440	0.162	0.72	0.66
(7) I feel proud when students make learning progress.	0.85	−4.134	0.167	0.74	0.65
(8) I feel touched by the support and understanding of the parents of the students.	0.81	−3.302	0.166	0.61	0.53
(9) I feel delighted when students enjoy my teaching support.	0.93	−3.744	0.167	0.59	0.49
(10) I feel excited when students are actively engaged in learning activities.	0.91	−3.828	0.167	0.63	0.56
**Anger**					
(11) I am annoyed when I am misunderstood by parents.	0.59	−0.089	0.147	1.05	1.10
(12) I am indignant when the society and/or public blame our teachers without any evidence.	0.75	−1.400	0.158	0.84	0.80
(13) I feel angry when I am treated unfairly (i.e., workload arrangement, salary level).	0.75	−1.400	0.158	0.97	0.92
(14) I feel angry when the society and/or public misunderstand our teachers.	0.84	−1.400	0.158	0.79	0.72
**Sadness**					
(15) I feel really sad when my students ?re up at me.	0.67	−0.712	0.153	1.09	1.09
(16) I feel disappointed when my school leaders ignore my efforts and contributions.	0.83	−1.562	0.158	0.71	0.66
(17) I feel frustrated when my promotion is stuck by stiff policies.	0.63	−1.388	0.157	0.91	0.87
(18) I feel disappointed when I do not get what I should get.	0.74	−1.487	0.158	0.94	0.90
**Stress**					
(19) I am worried about competition with my colleagues.	0.66	0.782	0.142	1.06	1.12
(20) I feel stressed by parents’ high expectations for teaching assistants.	0.71	−0.290	0.149	0.86	0.90
(21) I feel stressed in the face of work-life imbalance (too much time for work, too little time for family).	0.77	−0.246	0.148	0.95	0.98
(22) I feel stressed when time is tight and there is a lot of work to do.	0.74	−1.215	0.155	1.07	1.04
(23) I feel guilty when I don’t have enough time with my family	0.79	−0.696	0.152	0.96	0.96
(24) I am worried that students don’t take responsibility for their study.	0.58	−1.953	0.158	0.98	0.97

*, all item difficulties are in logits; SE, standard error; and MNSQ, mean square error.

For the four-item AIE scale, the CFA results suggested an excellent model fit for the unidimensional solution, such as CFI = 0.98, RMSEA = 0.09, SRMR = 0.03, and robust χ^2^ (2) = 5.199. All standardized factor loadings were significant (*p*-values < 0.001) and ranged from 0.56 to 0.87.

### Rasch Analysis

Table 3 shows the model comparison for the one-, two-, and five-dimensional models. The results selected the five-dimensional model to be the best model due to smaller values of AIC, BIC, and CAIC. The Rasch reliabilities for the five subscales were 0.81 (love), 0.83 (joy), 0.82 (anger), 0.86 (sadness), and 0.85 (stress), suggesting good reliabilities of these subscales. Table 2 presents detailed Rasch analysis results for the TEI under the five-dimensional model. Infit MNSQ identified all items as fitting items. We adopted the MNSQ values in the range of 0.6–1.4, which are commonly used for rating scales (e.g., Wright et al., 1994) to represent a good fit between data and model, Outfit MNSQ of four items (item 4 = 1.57, item 8 = 0.53, item 9 = 0.49, and item 10 = 0.56), and may be regarded as closing/marginal-fitting items. The reason why infit and outfit statistics sometimes vary is due to outfit statistics being sensitive to outliers, whereas infit is devised by down-weighting outliers and focusing more on the responses close to the item difficulty (or person ability). Also, the sample size might not have been large enough to yield a good detection of the fitting items (e.g., Wang and Chen, 2005). In addition, by investigating these items’ contents through content experts and ensuring they were all related to emotion, the MNSQs of the four items were very close to the critical range set for the rating scales (Wright et al., 1994). As a result, all items’ fit values were reasonable and acceptable for research purposes.

**TABLE 3:** Rasch analysis and model fit for the one- and five-dimensional models of the TEI.

Model	AIC	BIC	CAIC
One-factor solution	7961	8051	8078
Two-factor solution	7200	7296	7325
Five-factor solution	7001	7137	7178

*AIC, Akaike’s information criterion; BIC, Bayesian information criterion; CAIC, consistent AIC.*

**FIGURE 1:**
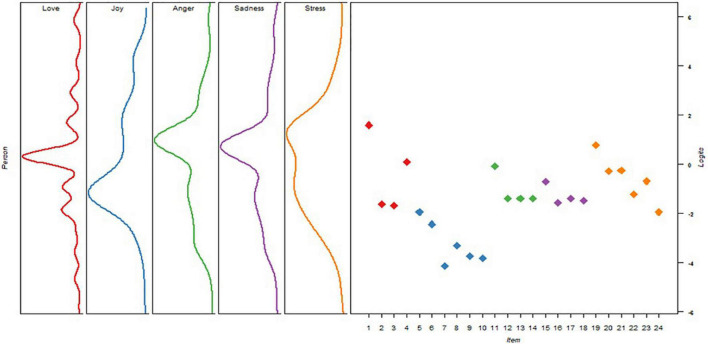
Item-person map of the TEI.

The item difficulties ranged from −4.13 to 1.59. The easiest item came from joy (*item 7: I feel proud when students make learning progress*), and the most difficult item came from love (*item 1: I love the profession of teaching assistant because unlike other professions, I can witness the growth and development of students with special educational needs*). One possible reason why the joy items are relatively easer for TAs would be because half of this subscale is about generating positive feelings from students’ learning outcomes. Assisting students with diverse abilities in achieving their learning outcomes are usually TAs’ regular practices/main duties (Chan et al., 2020; Webster and De Boer, 2021); therefore, TAs might not have difficulties in rating these items. For other items of assessing joy, they are about joyful feelings of having social support from colleagues and parents. Items on having social support and generating joyful feelings might not be difficult to rate. However, TAs’ difficulty in rating item 1 in love may be explained by the unstable features of their position (e.g., Webster and Blatchford, 2013). To love the job of a TA as an educational professional might be easy; however, to have a stable TA position to witness SEN students’ growth and development that usually requires a long period of time might not be easy for TAs based on challenges and unstable job features (e.g., Giangreco, 2013) (for details, refer to our review and introduction of the role of TAs in inclusive education in the section “Literature Review”). Figure 1 displays the item-person map (also called he Wright map), to present the hierarchy of the measures with respect to person abilities and item difficulties. The distributions of person abilities on each of the five continuums on the left side present the five subscales, indicating that those with higher levels of emotion were placed at the top of the continuum, and those with lower levels of emotion were placed at the bottom of the continuum. The distribution of item difficulty on the right side presents the higher item difficulties at the top and the lower ones at the bottom. One can find that the TEI provided a fairly targeted measurement of TAs’ emotions, though the range of item difficulty was smaller than that of person ability. Furthermore, the ordered nature for the category parameters (Eq. 1) was held according to Linacre’s (2002) guidelines, suggesting that the four-point rating scale was appropriate to all 24 items. That is to say, the step parameters of the four-point scale increased monotonically from –7.31, –1.38, to 1.72, although there was a relatively big difference between the last two-step parameters.

Likewise, Table 4 displays that both infit and outfit MNSQs identified all four items as fitting items for the AIE scale. The item difficulties ranged from –3.12 to 4.48. The easiest item was item 2 (*It is the basic right of SEN students to integrate SEN students into mainstream classes*), and the most difficult item was item 1 (*Inclusive education can provide equal learning opportunities for SEN students*). We also attach the item-person map (refer to Supplementary Appendix Figure 1) showing that the four-item AIE scale supplied a fairly targeted measurement of TAs’ AIE. In addition, the estimated step parameters (i.e., –6.28, –0.03, and 6.31) were ordered, indicating that the four four-point rating scale was suitable for the four items. Both CFA and Rasch analysis selected the five-factor model as the best model to the TEI data. External validity by using the four-item AIE scale also showed the five emotions were distinguishable.

**TABLE 4:** Summary of Rasch analysis for the AIE scale.

Item I think, in the current Hong Kong…	Standardized Factor Loadings	Item Difficulty*	SE	Infit MNSQ	Outfit MNSQ
(1) Inclusive education can provide equal learning opportunities for SEN students.	0.56	4.483	0.182	0.86	0.80
(2) It is the basic right of SEN students to integrate SEN students into mainstream classes.	0.69	–3.119	0.186	0.80	0.76
(3) Inclusive education embodies social justice and fairness.	0.87	–2.423	0.186	0.95	0.90
(4) Inclusive education is a sign of a civilised society.	0.85	–2.910	0.187	0.92	0.89

*, All item difficulties are in logits; SE, standard error; and MNSQ, mean square error.

### Teacher Emotion Inventory and Attitudes Toward Inclusive Education

**FIGURE 2:**
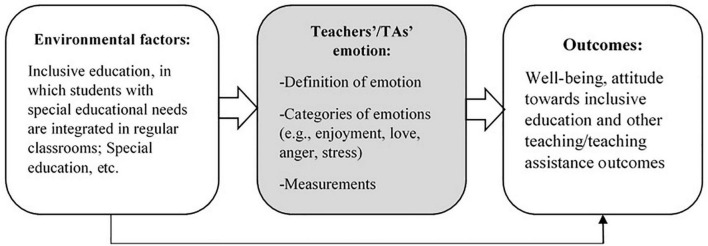
The conceptual framework of this study was designed by the first author in light of previous research (Pekrun, 2006; Chen, 2021).

**FIGURE 3:**
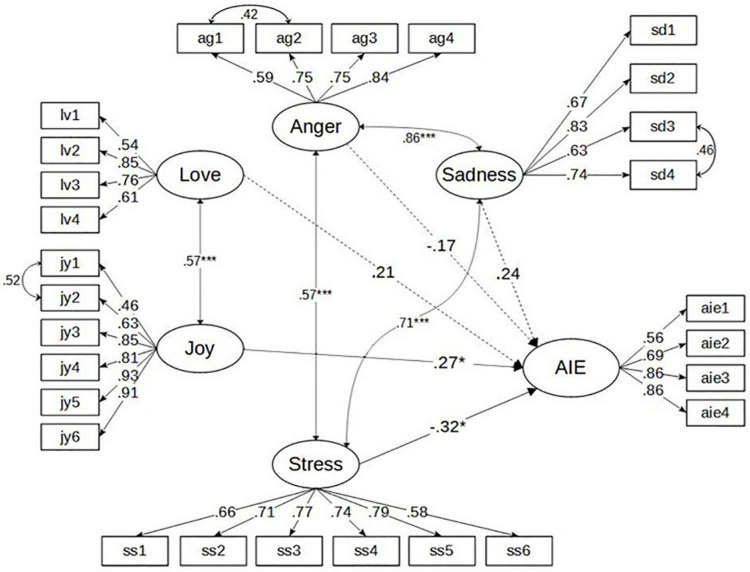
These dotted arrows mean non-significant paths. *p < 0.01, ***p < 0.001.

Table 5 shows that the correlation patterns between positive and negative emotions were reasonable: positive emotions positively correlated with each other, while negative emotions were positively correlated with each other. Positive emotions were negatively correlated with negative emotions. The correlations between AIE and five emotions were 0.29 (love), 0.32 (joy), −0.05 (anger), –0.03 (sadness), and –0.14 (stress), suggesting that TAs’ positive AIE were reasonably associated with their positive emotions and negatively correlated or almost uncorrelated with their negative emotions. Based on the conceptual framework shown in Figure 2, we performed SEM to explore the predictive roles of the five emotions in AIE. Demonstrated in Figure 3, the results showed that joy was a statistically significant positive predictor of AIE. Comparatively, stress was a statistically significant negative predictor of AIE. Path coefficients from love, anger, and sadness to AIE were not statistically significant.

**TABLE 5:** Correlations between the five subscales of the TEI and AIE.

Subscale	Love	Joy	Anger	Sadness	Stress	AIE
Joy	0.58*	–				
Anger	0.10	0.15*	–			
Sadness	0.04	0.10	0.64*	–		
Stress	0.16*	0.13	0.53*	0.62*	–	
AIE	0.29*	0.32*	−0.05	−0.03	−0.14*	–
Means (SD)	2.90(0.50)	3.23(0.44)	2.81(0.49)	2.85(0.50)	2.70(0.49)	2.92(0.42)
Min	1.50	1.00	1.00	1.00	1.00	1.50
Max	4.00	4.00	4.00	4.00	4.00	4.00
Skewness	−0.31	−0.36	−0.50	−0.46	−0.32	−0.55
Kurtosis	0.66	2.55	1.32	1.29	0.80	1.88

*p < 0.05, **p < 0.01.

## Discussion

This study extended previous TEI research with accumulated psychometric properties (Chen, 2016; Yang et al., 2021) to a sample of TAs working in Hong Kong. TA populations are usually requested to assist in SEN students’ learning and other teaching duties in inclusive education. Compared to teachers, this group of supporting staff is more likely to experience many more negative emotions from the challenges of supporting teachers and SEN students. Previous research also found that TAs are often misused and overused in inclusive education (Giangreco, 2013). Detailed results in relation to the six hypotheses based on the TEM (Figure 2) are discussed as follows.

### Within-Network Validity

The results adequately supported H1, which means the five-factor solution for the internal structure of TEI had the best fit supported by the results from the dual approach (Rasch and CFA) to perform item- and factor-level analyses. The results support the multicomponential features of emotions (e.g., Scherer, 2005; Fontaine et al., 2007; Frenzel et al., 2016), given that the one-factor and two-factor solutions of the emotion models had poor model fit compared to the five-factor solution. The results of the TEI in the new sample of TAs are consistent with the findings in the authors’ other validation study of special school teachers working in Hong Kong (Yang et al., 2021). Like their coworkers in special education, TAs working in inclusive education were also clearly differentiated in the five emotions.

### Between-Network Validity

H2 was supported given that the correlational pattern was consistent with this hypothesis: all three negative emotions were negatively correlated with AIE (Table 5). However, the negative correlations between two negative emotions (anger and sadness) and AIE were not statistically significant (*r* = −0.05 and *r* = −0.03, respectively). The results may indicate unimportant influences from the two negative emotions TA generated from contextual factors that can be conceptually differentiated (e.g., parents’ high expectation/misunderstanding of their work or difficulties they met in promotion) from their AIE as a platform to offer equal learning opportunities to SEN students (refer to Tables 2, 4 for item statements of the two scales). In addition, cultural values to emotion regulation in experiencing negative emotions in school settings (Yin and Lee, 2012) or “faking emotions” found in Western teachers (Burić and Frenzel, 2021) may partially explain this observation. For example, Yin and Lee (2012) found that Chinese teachers usually control their negative emotions for establishing a harmonious teaching-and-learning atmosphere to achieve teaching objectives. Sadness in this study also covered TAs’ negative emotion generated from students’ misbehaviors, e.g., “I feel really sad when my students fire up at me.” Yin and Lee (2012) summarized this kind of teachers’ suppression of negative emotions for achieving positive teaching outcomes as Chinese teachers’ emotional rules (see also Yin, 2016), while Burić and Frenzel (2021) described a similar phenomenon as “faking emotions” and found teachers’ “faking emotions can evidently be considered as beneficial for student engagement” (p. 347). Given the important role of TAs in supporting students with SEN who need more social and emotional caring compared to typically developed students (e.g., Giangreco, 2013; Chan et al., 2020; Webster and De Boer, 2021), possible suppression of negative emotions for harmonious school settings (known as social functions of emotion regulation; Fischer and Manstead, 2016, p. 424) or “faking emotions” (Burić and Frenzel, 2021) for achieving positive outcomes for SEN students when needed in inclusive education might not be unusual.

The path analysis provided additional support to the relation between positive emotions and AIE with an exception that the path coefficient from love to AIE was not statistically significant, although positive. Taking a closer look at the descriptions of love, joy, and AIE, we may provide some plausible explanations to the results. Love in the TEI describes TAs’ positive feelings regarding TAs as an educational professional (refer to Table 2 for detailed statements). Joy specifically describes TAs’ positive feelings of witnessing SEN students’ learning enjoyment, progress, engagement, etc. In other words, love is for working as a TA, while joy is mainly for seeing SEN students’ learning outcomes. Given that AIE in this study mainly referred to TAs’ attitude toward the implementation of inclusive education in Hong Kong, for the sake of SEN students’ equal learning opportunities, social justice, and fairness (SEN students-centered), it is reasonable to observe there is a much stronger link between TAs’ joy to witness SEN students’ gains from inclusive education and AIE than their love of TA as an educational professional (TAs’ identity-oriented) [refer also to Trent (2014)]. In this path analysis, we also found negative predictive roles of anger and stress. The negative predictive role of stress was statistically significant. No such result was found with anger. As noted earlier, positive attitudes toward implementing inclusive education to support SEN students might not necessarily or logically be linked with anger and sadness TAs generated from a range of experiences with colleagues, parents, and career promotion (refer to Table 2 for statements of anger and sadness). For future research, we would suggest to further explore the status of TAs’ emotional labor to “suppress” emotions for social functions (e.g., for maintaining/enhancing harmonious social relationships with colleagues, parents, and students). It is also meaningful to explore what role TAs’ emotion regulation strategies may play in adjusting the relationship between their experiences of negative emotions in work and AIE to support SEN students. This hypothesis needs to be verified by further replicated studies with TAs and teachers. We echo previous research’s arguments that teachers’ emotional displays play an important role in affecting students’ attitudes and behavior (e.g., Van Kleef et al., 2012) and in schools for positioning teachers themselves “as competent professionals” (Stark and Bettini, 2021). Given that not only positive but negative emotions teachers inevitably experience in teaching (e.g., Chen, 2016; Frenzel et al., 2016), teachers’ emotional displays determined by their emotional labor (i.e., to hide emotions, to fake emotions, to re-evaluate emotional antecedents) matter in teaching and learning. Given the highly demanding work to assist both school teachers and SEN students in teaching and learning (Giangreco, 2013; Chan et al., 2020), TAs’ emotional regulation strategies in work are worthwhile to be explored in future research. In this study, we did not find TAs’ anger and sadness as two significantly negative predictors of AIE and suspected their emotional regulation strategies and other reasons were causing their anger and sadness (Table 2) than inclusive education *per se*, which may influence the strength of the links between the two negative emotions and AIE.

## Theoretical and Empirical Implications

Both theoretical and empirical implications are highly related to the worrisome status of TAs internationally. The two reviews conducted by Giangreco et al. (2013) and see footnote text 1 could help us summarize what we reviewed in the “Introduction” and “Literature Review” sections. These scholars summarized that the utilization of TAs continues to increase in a variety of countries and regions (e.g., the United States, the United Kingdom, Australia, Ireland, New Zealand, Canada, Cyprus, Finland, Iceland, Italy, Norway, and Hong Kong). TAs continue to work with a wide range of students with SEN (e.g., there are nine types of SEN in Hong Kong), and they are engaged in various supporting roles (e.g., personal care, fostered students’ learning and inclusion, and instructional duties). These scholars (Giangreco et al., 2013; see footnote text 1), also observed research consistently found TAs often experience a wide range of challenges (e.g., low salary, low status, lack of job security, and stressful working conditions), affecting their emotions negatively and job stress, which may negatively cause problems for schools and students (e.g., high turnover and lower quality/unstable services).

Taking a close look at teachers’ positive emotions, researchers have consistently found teachers’ positive emotions are associated with teachers’ wellbeing (Fried et al., 2015; Chen, 2016) and a wide range of students’ learning outcomes [Hagenauer et al., 2015; refer to Chen (2021) for a systematic review]. Despite a growing number of studies on teachers’ emotions, for example, in Chen’s (2021) systematic review of publications during 1985–2019 (with over 800 articles on teacher emotions), unfortunately, there was not a single study conducted on TAs. This study enriches the current literature of teachers’ emotions by adding TAs’ data. This study found support to the appropriateness of the extended TEM (Figure 2) in its use with TAs. The findings enrich teacher emotion research by adding that the multidimensional construct of teacher emotion is also applicable to TAs. The positive and statistically significant correlations between positive emotions and TAs’ AIE extend the current research by typically examining the role of teacher self-efficacy in influencing teachers’ AIE (Yada et al., 2022). The TEM illustrated in Figure 2 can serve as a conceptual framework for extending the current scope of studying either self-efficacy or emotions in teachers to TAs for enriching not only teacher emotion research but also finding solutions to supporting SEN students’ social, emotional and academic outcomes (Yang et al., 2015) with professional assistance from the population of TAs.

Empirically, given the absence of a validated instrument to assess TAs’ emotions, this study has filled this research gap by providing this validated version. Researchers and educators can use it further with Chinese teachers and TAs for assessment and evaluation purposes in order to evaluate the effects of professional training on teachers and TAs who need to support SEN students. The positive correlations between TAs’ positive emotions and their AIE also have implications to professional training, to support this group of supporting staff in inclusive education. Given the increasing number of SEN students in mainstream secondary schools (*n* = 22, 380 in the 2017/2018 school year) as documented by the Legislative Council of Hong Kong^3^, caring for TAs’ emotions matters to their job stratification, and contributes to assisting SEN students. In terms of a compelling need to support TAs, we also noticed 85% out of the 204 TAs in this study reported that they have not received any professional training. We applaud Chan et al.’s (2020, p. 9) calling.

To better equip TAs with more skills for future needs, to provide support for SEN students, and to enhance TAs’ engagement in more complex classroom duties, professional development programs for TAs may have to be included to enable them to have more hands-on experience in workplace.

## Limitations and Future Directions

This study has its meaningful and timely contribution to a validated instrument of TEI in TAs. However, its cross-sectional design cannot allow readers to infer a causal relationship between TAs’ emotions and AIE. For future research, it would be promising to consider longitudinal designs in order to test whether there is a reciprocal relationship between teacher emotions and AIE. Longitudinal research would also be helpful for researchers and educators to examine the developmental trend of TAs’ emotions. Based on the conceptual framework (Figure 2), future research that includes comparative groups of teachers is also needed in order to shed more insights into understanding the key features of TAs’ emotions. Future studies may increase sample sizes and recruit comparative samples in special education in both Eastern and Western contexts to examine the incremental validity of the TEI. Researchers can also take the disability type(s) of students TAs’ support into account for understanding their emotions. As noted earlier, teachers may experience more negative emotions in supporting some types of SEN students compared to others. For example, Ruble et al. (2011) found that teachers experience more negative emotions influenced by the behavioral problems of students with autism.

Future studies can also consider experimental designs for tracing the developmental trend of TAs’ emotions in inclusive education before and after professional training to TAs. Comparative research between teachers and TAs who need to support SEN students is also promising in order to bring about more insights to understand both groups of educators’ emotions. Based on these empirical pieces of evidence, governments and higher education institutions can cooperate in developing tailor-made professional training schemes to not only teachers but also TAs for them to boost their professional development [refer also to Trent (2014)] and support SEN students in inclusive education (Rose and Forlin, 2010). In urging an agenda of research on TAs among international scholars, Webster and De Boer (2021) critically commented:

Given the pre-eminence of TA deployment as a means to facilitate access to and participation in mainstream education for pupils with special educational needs, we argue that the continued lack of large-scale data on TAs’ characteristics, experiences, practices and impact poses a risk to advancing the global inclusion agenda (p. 294).

We echo this urgent research agenda on TAs. To the best of our knowledge, this is the first study to assess TAs’ emotions experienced in inclusive education. Together with another validation study of special school teachers (Yang et al., 2021), the research team has extended previous work to make the TEI available (Table 3) for international scholars’ use. The parsimonious scale (four items) of AIE, which has been used in 122 (about 31%) of Hong Kong secondary schools with inclusive classes, would also be helpful for researchers to use in their educational settings for verifying incremental validity and comparing purposes across cultures in terms of AIE. We hope that this study, together with its findings, will facilitate many more empirical studies among TAs by including more variables in the TEM (Figure 2) so as to enrich the picture of understanding TAs and develop efficient training programs for productive emotional support to this relatively vulnerable group of educational professionals (Trent, 2014; Webster and De Boer, 2021).

## Conclusion

Despite the crucial assisting role of TAs in inclusive schools, there are no focused studies on assessing and understanding their emotions in supporting regular teachers and SEN students. Based on previous TEI research (Chen, 2016; Yang et al., 2021) on teachers, this study extends to TAs who need to support SEN students in inclusive education in Hong Kong. Both CFA and Rasch analysis supported the five-factor internal structure. The result is consistent with previous research (e.g., Yang et al., 2021). Using a measure of TAs’ AIE as an external variable, we tested the external validity of TEI. We found that positive emotions (love and joy) are positively associated with TAs’ AIE. In contrast, negative emotions (anger, anxiety, and stress) are almost uncorrelated or negatively correlated with TAs’ attitudes. The pattern supports the differentiation of discrete emotions among TAs. To support TAs’ positive emotional experiences in inclusive education, we encourage more quantitative or mixed-method studies for understanding and helping TAs’ emotional wellbeing to make the most of their supporting work in inclusive schools.

## Data Availability Statement

The original contributions presented in the study are included in the article/Supplementary Material, further inquiries can be directed to the corresponding author.

## Ethics Statement

The studies involving human participants were reviewed and approved by the Human Research Ethics Committee (HREC), The Education University of Hong Kong. The patients/participants provided their written informed consent to participate in this study.

## Author Contributions

LY proposed the study, conceptualized, designed the survey study, co-collected the data, and wrote, revised, and finalized the complete draft. C-LH contributed to Rasch analysis and wrote the results. TY contributed to CFA analysis and wrote the results. KS co-conceptualized this study and contributed to data collection and resources for the manuscript. All authors contributed to the article and approved the submitted version.

## Conflict of Interest

The authors declare that the research was conducted in the absence of any commercial or financial relationships that could be construed as a potential conflict of interest.

## Publisher’s Note

All claims expressed in this article are solely those of the authors and do not necessarily represent those of their affiliated organizations, or those of the publisher, the editors and the reviewers. Any product that may be evaluated in this article, or claim that may be made by its manufacturer, is not guaranteed or endorsed by the publisher.

## Footnotes

1 Yang, L., Lee, J. C.-K., Zhang, D., and Chen, J. (under review). Examining the relationships among teaching assistants’ self-efficacy, emotional well-being and job satisfaction.

2
https://www.gov.hk/en/about/abouthk/factsheets/docs/education.pdf

3
https://www.legco.gov.hk/research-publications/english/1819issh22-special-educational-needs-20190219-e.pdf
