# Supplementary material for: Assessing Emotions of Teaching Assistants in Inclusive Education
Source: Front Psychol. 2022 Jul 14;13:813726. doi: 10.3389/fpsyg.2022.813726 (PMC9331288; doi:10.3389/fpsyg.2022.813726)

**Appendices**

**Table A1**

*Demographic information of seven validation studies on teacher emotions*

| Studies | Instrument | Items in total | No. of Discrete emotions | | Teachers  (Sample size) | Regions/ countries | Educational setting | Validation methods |
| --- | --- | --- | --- | --- | --- | --- | --- | --- |
|  |  |  | PEs | NEs |  |  |  |  |
| Chen (2016) | Teacher Emotion Inventory (TEI) | 26 | 2 | 3 | 2084 | Mainland China &  Hong Kong SAR | Mainstream primary education | EFA & CFA |
| Atmaca et al. (2020) | Chen’s (2016) TEI | 26 | 2 | 3 | 564 | Turkey | Mainstream preschool to high schools | CFA |
| Yang et al. (2021) | Chen’s (2016) TEI | 25 | 2 | 3 | 66 | Hong Kong SAR | Special education (secondary schools) | Rasch analysis |
| Frenzel et al. (2016) | Teacher Emotion Scales(TES) | 12 | 1 | 2 | 414 | Germany & Canada | Mainstream  primary and secondary education | CFA |
| Hong et al. (2016) | Achievement Emotions Questionnaire (AEQ-T) | 20 | 2 | 3 | 150 | Japan | Mainstream primary and secondary education | CFA |
|  |  |  |  |  | 208 | Korea |  |  |
| Burić et al. (2018) | Teacher Emotion Questionnaire (TEQ) | 36 | 3 | 2 | 2345 | Croatia | Mainstream primary and secondary education | CFA |
| Gramipour et al. (2019) | Teacher Academic Emotion (TAE) Scale | 54 | 3 | 5 | 114 | Tehran | Mainstream secondary education | CFA |

*Note.* EFA = Exploratory Factor Analysis; CFA = Confirmatory Factor Analysis; PEs = positive emotions; NEs=negative emotions

**Table A2**

*Confirmatory factor analysis and model fit indices of the AIE*

| Models | CFI | RMSEA | SRMR | Robust χ^2^ | AIC | BIC |
| --- | --- | --- | --- | --- | --- | --- |
| One-factor solution | .981 | .089 | .025 | 5.199 | 916 | 956 |

**Figure A1**

*Item-person map Item fit of the scale of attitude to inclusive education (AIE)*


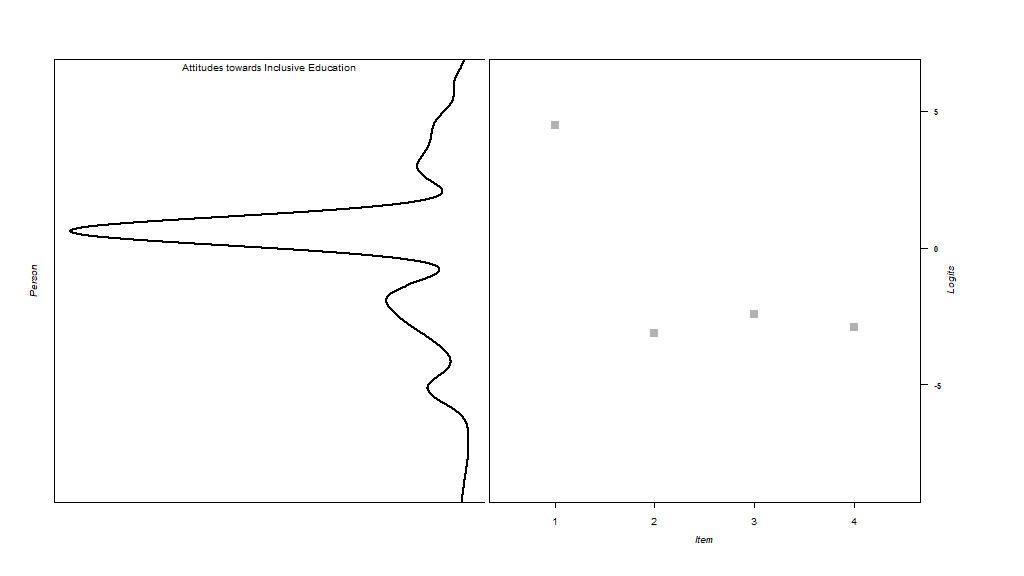

Supplement: Supplementary file 1 [file Data_Sheet_1.docx]
